# P-1083. Antimicrobial Activity of Aztreonam-Avibactam and Comparator Agents against a Large Collection of *Stenotrophomonas maltophilia* Isolates Collected in United States Medical Centers (2019-2023)

**DOI:** 10.1093/ofid/ofae631.1271

**Published:** 2025-01-29

**Authors:** Helio S Sader, Timothy Doyle, Marisa Winkler, S J Ryan Arends, Mariana Castanheira

**Affiliations:** JMI Laboratories, North Liberty, Iowa; Element Materials Technology/Jones Microbiology Institute, North Liberty, Iowa; Element Materials Technology/Jones Microbiology Institute, North Liberty, Iowa; JMI Laboratories / Element, North Liberty, Iowa; JMI Laboratories, North Liberty, Iowa

## Abstract

**Background:**

The occurrence of *S. maltophilia* infections has increased continuously in the last few years. We evaluated the *in vitro* activities of aztreonam-avibactam (ATM-AVI) and comparators against a large collection of *S. maltophilia* clinical isolates.

Activity of aztreonam-avibactam and comparator agents stratified by infection type.

**Figure:**
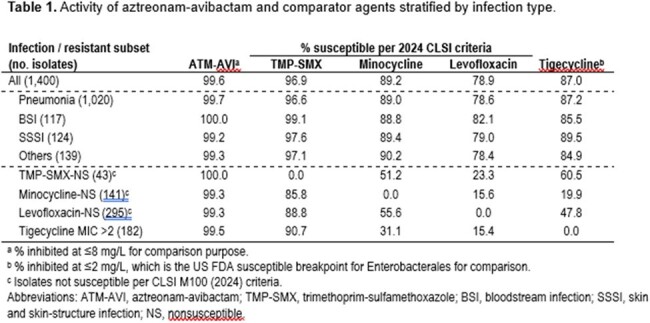

**Methods:**

1,400 clinical isolates were consecutively collected from 62 United States (US) medical centers. Infection sites included pneumonia (n=1,020), bacteremia (n=117), skin and skin-structure infection (SSSI; n=124), and others (n=139). Isolates were susceptibility tested by CLSI M07 broth microdilution methods. ATM-AVI was tested with AVI at fixed 4 mg/L and a pharmacodynamic/pharmacokinetic susceptible (S) breakpoint of ≤ 8 mg/L was applied for comparison.

**Results:**

ATM-AVI inhibited 99.6% of isolates at ≤ 8 mg/L (MIC_50/90_, 2/4 mg/L) and demonstrated potent activity against isolates from all infection types (Table). ATM-AVI inhibited 99.7% of isolates from pneumonia and 100.0% of isolates from BSI at ≤ 8 mg/L and retained potent activity against isolates non-S to other agents commonly used to treat *S. maltophilia* infections. ATM-AVI was active (MIC ≤ 8 mg/L) against 100.0% of trimethoprim-sulfamethoxazole (TMP-SMX)-non-S isolates, 99.3% of isolates non-S to minocycline or levofloxacin, and 99.5% of isolates with tigecycline MIC > 2 mg/L. Moreover, ATM-AVI activity remained stable during the study period; the percentages inhibited at ≤ 8 mg/L varied from 100.0% in 2019 and 2020 to 99.3% in 2023. TMP-SMX (MIC_50/90_, ≤ 0.12/0.5 mg/L; 96.9% S) also showed good activity against *S. maltophilia*. Minocycline (MIC_50/90_, 0.5/2 mg/L) and levofloxacin (MIC_50/90_, 1/8 mg/L) were active against 89.2% and 78.9% of isolates according to the 2024 CLSI breakpoint criteria.

**Conclusion:**

ATM-AVI exhibited potent activity and broad coverage against *S. maltophilia* from US hospitals and its activity was not adversely affected by resistance to other agents. Our results indicated that ATM-AVI may represent a valuable option to treat *S. maltophilia* infections, addressing a major unmet medical need.

**Disclosures:**

**Marisa Winkler, MD, PhD**, Element Iowa City (JMI Laboratories) was contracted to perform services in 2023 for > 30 biotech and pharmaceutical companies: Grant/Research Support

